# Traditional Chinese Medicine Formula *Kang Shuai Lao Pian* Improves Obesity, Gut Dysbiosis, and Fecal Metabolic Disorders in High-Fat Diet-Fed Mice

**DOI:** 10.3389/fphar.2020.00297

**Published:** 2020-03-25

**Authors:** Shuqing Gong, Tingting Ye, Meixia Wang, Mengying Wang, Yufei Li, Lina Ma, Yulian Yang, Yi Wang, Xiaoping Zhao, Li Liu, Min Yang, Huan Chen, Jing Qian

**Affiliations:** ^1^Pharmaceutical Informatics Institute, College of Pharmaceutical Sciences, Zhejiang University, Hangzhou, China; ^2^Key Laboratory of Microbial Technology and Bioinformatics of Zhejiang Province, Zhejiang Institute of Microbiology, Hangzhou, China; ^3^NMPA Key Laboratory for Testing and Risk Warning of Pharmaceutical Microbiology, Zhejiang Institute of Microbiology, Hangzhou, China; ^4^Chronic Disease Research Institute, Department of Nutrition and Food Hygiene, School of Public Health, Zhejiang University School of Medicine, Hangzhou, China; ^5^School of Public Health, Zhejiang University School of Medicine, Hangzhou, China; ^6^College of Preclinical Medicine, Zhejiang Chinese Medical University, Hangzhou, China; ^7^Technical Center, Chiatai Qingchunbao Pharmaceutical Co. Ltd, Hangzhou, China

**Keywords:** Traditional Chinese medicine, high-fat diet, obesity, gut microbiota, fecal non-targeted metabolomics, correlation analysis

## Abstract

High-fat diet (HFD)-induced obesity is a risk factor for many metabolic disorders including cardiovascular diseases, diabetes, and fatty liver disease. Although there are accumulating evidences supporting the assumption that regulating gut microbiota as well as its metabolic status is able to mitigate obesity, the inner relationship between the obesity-related gut microbiota and the relevant metabolites are not well defined. In current study, we applied a traditional herbal formula *Kang Shuai Lao Pian* (KSLP) to HFD-fed mice and evaluated its effect against obesity. Emphases were addressed on identifying profiles of gut microbiota and fecal metabolites with the aid of 16S rRNA gene sequencing and non-target fecal metabolomics techniques. We showed that KSLP could improve HFD-induced obesity, glucose tolerance disorder, as well as gut dysbiosis. In the gut, KSLP corrected the increased abundance of Firmicutes and Proteobacteria, increased ratio of Firmicutes/Bacteroidetes, and decreased abundance of Bacteroidetes caused by HFD. KSLP also reversed HFD-induced significant changes in the abundance of certain genus including *Intestinimonas*, *Oscillibacter*, Christensenellaceae_R-7_group, Ruminococcaceae_UCG-010, and *Aliihoeflea*. Pearson correlation analysis indicated that except for Ruminococcaceae_UCG-010, other four genera had positive correlations with obesity. In addition, 22 key fecal metabolites responding to KSLP treatment were identified. Pearson correlation analysis showed that those metabolites are intimately related to KSLP effective genera of *Intestinimonas*, *Oscillibacter*, and Christensenellaceae_R-7_group. Our results indicate that KSLP is a promising traditional Chinese medicine (TCM) applicable for individuals with HFD habit. *Intestinimonas*, *Oscillibacter*, and Christensenellaceae_R-7_group might be responsible for the regulatory effect of KSLP. Linking of obesity phenotypes with gut microbiota as well as fecal metabolites is therefore a powerful research strategy to reveal the mechanism of obesity and the targets of intervention.

## Introduction

Defined as a disease status related to various health problems and reduced life span (Hoyt et al., 2014), obesity has become a serious health issue in the past decades (James, 2008). It is estimated that more than 1 billion people are prone to be obese by the year 2030 worldwide (Kelly et al., 2008). Due to the accumulation of adipose tissue, obese individuals appear susceptible to metabolic disorders including insulin resistance, type 2 diabetes, fatty liver disease, and cardiovascular diseases (Kopelman, 2000). To date, there is still a lack of promising strategies for the prevention and treatment of obesity, partially due to the limited understanding of the mechanisms controlling the occurrence of obesity and the development of its related metabolic diseases.

Among the complex environmental and genetic factors, gut microbiota plays a critical role in regulating HFD-induced obesity and obesity-related metabolic diseases (Turnbaugh et al., 2006; Cani et al., 2009; Ridaura et al., 2013). It is recognized that diet has approximately 57% influence on the gut microbiota structure while genetic factors has approximately 12% (Tomasello et al., 2014). In line with clues that the occurrence of obese phenotype is associated with gut microbiosis characterized by richer abundance of Firmicutes and poorer Bacteroides in genetic obese ob/ob mice (Ley et al., 2005) and that transplantation of gut microbiota of human obese twins into mice fed with low-fat diet results in transmissible adiposity phenotypes (Ridaura et al., 2013), targeting the structure and function of gut microbiota could be a promising strategy for the prevention and treatment against obesity.

Gut microbiota affects nutrient acquisition, energy harvest, and metabolic pathways of the host (Devaraj et al., 2013; Le Chatelier et al., 2013). In the scenario of obesity, disorders of lipid, carbohydrate, bile acid, and amino acid metabolism are all likely related to compositional changes in gut microbiota (Caesar et al., 2010; Yokota et al., 2012; Devaraj et al., 2013; Neis et al., 2015). The investigation of fecal metabolomics, especially its association with the functional readout of gut microbiome, is of great value for the understanding of diet–microbiota–metabolism interactions (Cheng et al., 2018; Zierer et al., 2018).

Chinese herbal medicine has shown powerful capacity of improving both obesity and its metabolic diseases through the regulation of gut microbiota (Hasani-Ranjbar et al., 2009). Indeed, as gut microbiota composition is essential for the occurrence of obesity, varieties of bioactive substances from herb medicine were revealed to have positive impacts on gut microbiota and therefore could be used for the prevention or treatment of lipid metabolic disorders (Yu et al., 2018; Zhang et al., 2019b). For example, it is recently reported that the therapeutic effect of ganoderma lucidum against HFD-inducing obesity is actually working through the compositional regulation of gut microbiota (Chang et al., 2015; Chang et al., 2017). *Kang Shuai Lao Pian* (KSLP) is a famous traditional Chinese medicine (TCM) formulated from a court prescription of the Ming Dynasty. It comprises *Rehmannia glutinosa* (Gaertn.) DC., *Panax ginseng* C.A.Mey., *Asparagus cochinchinensis* (Lour.) Merr., *Ophiopogon japonicus* (Thunb.) Ker Gawl., *Lycium chinense* Mill., and *Poria cocos* (Schw.) Wolf. In China, it is a widely used health care product for delaying senescence. The application of KSLP could improve learning and memory but inhibit brain lipid peroxidation in D-galactose-induced aged rats (Tao, 2009). Although a direct link between HFD-induced obesity and KSLP has never been reported, there were clinical observations indicating people with long-term KSLP administration prone to have a lean appearance. Besides, we have revealed that while taking KSLP as food supplement for 6 weeks, mid-aged mice appeared to have relatively less white but more brown adipose tissue distribution (Gong et al., 2020). Therefore, it is worthwhile to investigate whether KSLP has any interventional effect on HFD-induced obesity. In the current study, we established an HFD-induced obesity mouse model and applied KSLP as an intervention. In addition to the assessments of body weight, adipose tissue distribution, and blood metabolites chemistry, 16S rRNA gene sequencing and fecal non-targeted metabolomics studies were apparently performed.

## Materials and Methods

### KSLP Preparation

KSLP (Med-drug permit no. B20021021) was obtained from Chiatai Qingchunbao Pharmaceutical Co. Ltd. (Hangzhou, China). Composed of six herbs including *R. glutinosa* (Gaertn.) DC., *P. ginseng* C.A.Mey., *A. cochinchinensis* (Lour.) Merr., *O. japonicus* (Thunb.) Ker Gawl., *L. chinense* Mill., and *P. cocos* (Schw.) Wolf. with the mixed proportion of the respective compound being 409:167:26:26:26:77, the preparation of KSLP is followed as described in the patent (CN 1943707 B) (Zhou, 2012). The HPLC fingerprint results from three different batches (Batch 20171020, 20180302, and 20180716) were listed (**Supplementary Figure 1**). In the current study, KSLP (Batch 20171020) was dissolved in Milli-Q water and prepared as drug resuspension before use.

### Animals and Experimental Design

C57/BL6 mice (male, 6–8 weeks) were purchased from the Shanghai ReMed Biotechnology Co. Ltd. (Shanghai, China, www.remed-bio.com) and maintained under the following environment: 24–26°C, 40–60% humidity, 12-h light/dark cycle, food and water ad libitum. All of the animal experiments were performed at Zhejiang Province center for disease control and prevention. The Guide for the Care and Use of Laboratory Animals was followed (Mason and Matthews, 2012).

Mice were divided into four groups: normal diet (ND, *n* = 5), normal diet supplied with KSLP (ND_K, *n* = 5), high-fat diet (HFD, *n* = 9), and HFD supplied with KSLP (HFD_K, *n* = 9). The normal diet feed was supplied by Zhejiang experimental animal center and the implementation of the standard was GB14924.1-2001. The HFD feed was supplied by Research Diets (New Brunswick, NJ, USA) with the energy supply ratio being 60% fat, 20% protein, and 20% carbohydrate. Mice were supplied with either KSLP resuspension at 0.45 g/kg/day or the same amount of water by intragastric administration for 12 consecutive weeks, respectively.

The body weight of mice and food intake were measured every week. On the 9th week of the experimental period, oral glucose tolerance test (OGTT) was performed. At the end of the experiment, mice were fasted for 4 h before the eye blood was collected. Afterwards, samples of mice were collected for further studies. In detail, samples of visceral adipose tissue (VAT, including epididymal adipose, perirenal adipose, and mesentery adipose), subcutaneous adipose tissue (SAT), interscapular brown adipose tissue (BAT), and gastrocnemius muscles were extracted for weighting; intestine feces were collected and stored at −80°C till sent for 16S rRNA gene sequencing and non-targeted metabolomics analyses.

### Blood Biochemistry Test

Blood samples were kept still for 2 h before they were centrifuged at 3000 rpm for 15 min at 4°C. After centrifugation, the supernatants were collected for blood biochemistry test. Parameters including triglyceride (TG), total cholesterol (CHOL), low-density lipoprotein (LDL), high-density lipoprotein (HDL), and glucose were assayed on a Hitachi 7180 automatic biochemical analyzer (Kyoto, Japan).

### OGTT

For OGTT, mice were fasted for 4 h before they were intragastrically administered with glucose solution at a dosage of 2.5 g/kg. At baseline (0 min), 30, 60, and 120 min post glucose administration, tail blood was collected for the glucose level measuring (Super glucocard II GT-1640, ARKRAY Factory, Inc., Kyoto, Japan). A glucose curve was created and the area under the curve of glucose (AUC) was calculated according to the formula: AUC = (FPG/2 + 60 min PG + 120 min PG/2) × 1 h mmol·h/L (FPG: fasting plasma glucose).

### Gut Microbiota Sequencing and Data Analysis

Parts of fecal samples were sent to Zhejiang Tianke high-tech Co., Ltd (Hangzhou, China. http://www.tkgeneclub.com/tkgeneclub/index.html) for 16S rRNA gene sequencing. Total genomic DNA was extracted from 0.25 g of feces using the PowerSoil^®^ DNA Isolation Kit (MO BIO, Cat. No. 12888, Carlsbad, CA, USA) according to the manufacturer’s protocols. DNA concentration and purity were monitored on 1% agarose gels. According to the concentration, DNA was diluted to 1 ng/μl using sterile water. 16S rRNA genes were amplified used specific primer (16S V3–V4: 341F-806R) with the barcode. All PCR reactions were carried out with KAPA HiFi™ HotStart ReadyMix (2×). The PCR product was confirmed by using 1% agarose gel electrophoresis. The amplified products were purified with Beckman DNA Clean Beads and quantified by the Qubit 2.0 fluorometer (Invitrogen, Carlsbad, CA, USA). The library was diluted to 60 pM. The enriched library was loaded in Ion 530™ Chip and sequenced on an Ion S5™ platform (Thermo Fisher Scientific, Waltham, MA, USA) and about 500-bp single-end reads were generated. Raw sequencing data are available at the Sequence Read Archive (SRA) database of NCBI and connected to bioproject PRJNA565488.

Single-end reads were assigned to samples based on their unique barcode and truncated by cutting off the barcode and primer sequence. Quality filtering on the raw reads were performed under specific filtering conditions to obtain the high-quality clean reads according to the Cutadapt (https://cutadapt.readthedocs.io/en/stable/). The tags were compared with the reference database (Gold database, http://drive5.com/uchime/uchime_download.html) using UCHIME algorithm (UCHIME Algorithm, http://www.drive5.com/usearch/manual/uchime_algo.html) (Edgar et al., 2011) to detect chimera sequences and then the chimera sequences were removed (Haas et al., 2011). Then, the effective Tags were finally obtained. Operational Units (OTUs) were clustered with 97% similarity cutoﬀ using Uparse (Uparse V8.1.1861, http://drive5.com/uparse/) (Edgar, 2013). Representative sequence for each OTU was screened for further annotation through the SILVA reference database (http://www.arb-silva.de/) (Quast et al., 2013) by uclust (http://drive5.com/usearch/manual/uclust_algo.html) (Edgar, 2010) at 90% threshold. Principal coordinates analysis (PCoA) was displayed by vegan package in R software (Version 3.2.2). The ACE index, Chao1 index, Shannon index, Simpson index, and rarefaction curve were calculated with QIIME (Version 1.9.1) and displayed with R software (Version 3.2.2). The ACE index, Chao1 index, Shannon index, and Simpson index used Wilcoxon rank-sum test for statistical significance comparison among groups. Significant differences in genus levels between two groups were calculated using R software for *t* test to obtain *p* values. Linear discriminant analysis (LDA) effect size (LEfSe) (http://huttenhower.sph.harvard.edu/galaxy) combined with the standard tests (Kruskal–Wallis rank-sum test and Wilcoxon rank-sum test) with linear discriminate analysis was used for statistical significance comparison.

### Gene Expression Analysis

The expression of Akkermansia was further analyzed using real-time qPCR as described elsewhere (Everard et al., 2013). The primers were ordered from Sangon Biotech (Shanghai, China) and 16S rDNA was amplified as an endogenous reference gene. The relative expression was calculated with the ΔΔCT method and expressed as the fold change in comparison to the control (ND group).

### Non-targeted Metabolomics Study

Parts of fecal samples were sent to Metabolon in cooperation with Calibra Diagnostics, Ltd. (Hangzhou, China, www.metabolon.com) for non-targeted metabolomics study. In brief, approximately 50 mg of feces was freeze-dried in a freeze drier (FreeZone 2.5L, LABCONCO, Kansas City, MO, USA) for 8 h. Afterwards, the samples were subjected to a MicroLab STAR^®^ system (Hamilton, Switzerland) for automatic processing, which includes filtration, adding methanol to precipitate protein, and centrifugation before the supernatant of each sample was transferred to Evaporation System TurboVap^®^ II for organic solvent removing and mobile phase reconstitution.

Ultrahigh Performance Liquid Chromatography–Tandem Mass Spectroscopy (UPLC-MS/MS): All methods utilized a Waters Acquity ultraperformance liquid chromatography and a Thermo Scientific Q-Exactive high-resolution/accurate mass spectrometer interfaced with a heated electrospray ionization (HESI-II) source and Orbitrap mass analyzer operated at 35,000 mass resolution. The concrete conditions of UPLC and MS as well as the method validation were described in detail in previous articles (Long et al., 2017).

Compounds were identified by automated comparison of the ion features in the experimental samples to a reference library of chemical standard entries that included retention time (RI), molecular weight (m/z), and chromatographic data (including MS/MS spectral data) on all molecules present in the library developed at Metabolon (Evans et al., 2009; Dehaven et al., 2010). Furthermore, biochemical identifications are based on three criteria: retention index within a narrow RI window of the proposed identification, accurate mass match to the library ± 10 ppm, and the MS/MS forward and reverse scores between the experimental data and authentic standards. The MS/MS scores are based on a comparison of the ions present in the experimental spectrum to the ions present in the library spectrum. While there may be similarities between these molecules based on one of these factors, the use of all three data points can be utilized to distinguish and differentiate biochemicals. More than 3300 commercially available purified standard compounds have been acquired and registered into LIMS for analysis on all platforms for determination of their analytical characteristics. Peaks were quantified using area under the curve. For studies spanning multiple days, a data normalization step was performed to correct variation resulting from instrument inter-day tuning differences. Essentially, each compound was corrected in run-day blocks by registering the medians to equal one (1.00) and normalizing each data point proportionately. ANOVA contrasts were used to identify biochemicals that differed significantly between experimental groups.

### Statistical Analysis

Data are presented as mean ± standard errors of the means (S.E.M.). Statistical differences were assessed over two groups by analysis of variance (ANOVA) using GraphPad Prism 7.0 software. In metabolomics research, statistical analyses are performed in ArrayStudio; Student’s *t* test was performed between two groups, while ANOVA was used among over two groups. A *p* value less than 0.05 indicated statistical significance. Pearson correlation analysis was performed using SPSS and the corresponding heatmap was visualized using Excel and Adobe Illustrator.

## Results

### KSLP Decreased Body Weight, White Adipose Tissue Ratio, and Improved Glucose Tolerance Disorder in HFD Mice

**Figure 1:**
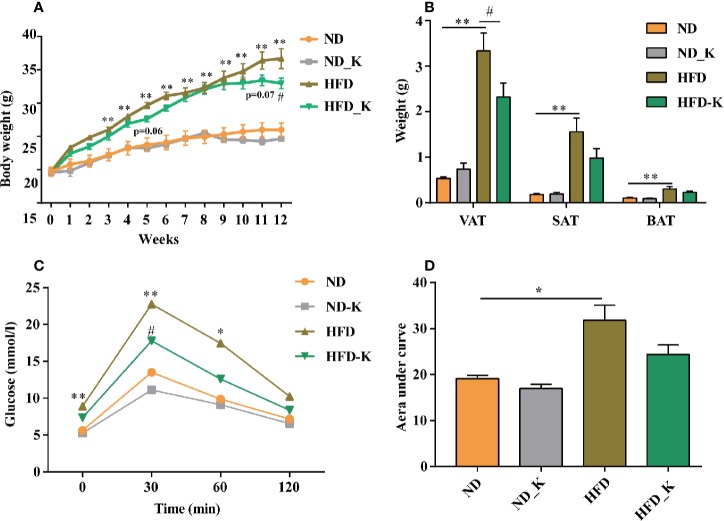
KSLP reduced body weight and improved the glucose tolerance in HFD mice. **(A)** The changes of body weight during the experimental course. **(B)** The effects of HFD and KSLP on visceral adipose tissue (VAT), subcutaneous adipose tissue (SAT), and brown adipose tissue (BAT). **(C)** OGTT after 9 weeks of KSLP treatment. **(D)** AUC of each group was calculated during the oral glucose tolerance test. Compared with ND group, *p < 0.05, **p < 0.01. Compared with HFD group, ^#^*p* < 0.05.

In the first panel of assessments, we compared the body weight, fat tissue distribution, and blood metabolic parameters among the groups of normal diet (ND), normal diet supplied with KLSP (ND_K), HFD, and HFD supplied with KSLP (HFD_K). As indicated in body weight curve (**Figure 1A**), ND group mice had slight body weight gain over the period of experiment, so did the ND_K group mice (*p* > 0.05, ND vs. ND_K). On the contrary, compared with ND mice, HFD group mice had a higher body weight gain speed. Starting from 3 weeks after HFD, the HFD mice had significant body weight gain as compared to ND mice (*p* < 0.05). The application of KSLP relieved the HFD-induced body weight gain. At the end of the experiment (12 weeks), the reduced body weight of HFD-K mice as compared to HFD were of statistical significance (*p* < 0.05). The inhibited body weight growing was not due to the inhibited appetite since the food intake between HFD and HFD_K groups had no significant difference (**Supplementary Figure 2**).

At the end of the experiment, samples of VAT (including epididymal adipose, perirenal adipose and mesentery adipose), SAT, and interscapular BAT were extracted and weighted for comparison. As indicated in **Figure 1B**, compared with ND mice, HFD mice had significant higher amounts of visceral adipose, subcutaneous adipose, as well as interscapular brown adipose (*p* < 0.05). KSLP reduced the net weight of these three types of fat tissues especially the visceral adipose (0.9858 ± 0.2058 vs. 1.559 ± 0.3042 g, *p* < 0.05). Notably, the reduced overall body weight of KSLP on HFD mice was not at the cost of muscle since KSLP treatment had no significant effect on the muscle weight of mice (**Supplementary Figure 3**).

Next, we detected lipid and glucose level (TG, CHOL, HDL, LDL, and glucose) for each mouse. As indicated in **Table 1**, except for the reduced level of TG (*p* < 0.05), KSLP had no effect on CHOL, HDL, LDL, and blood glucose on ND mice. As expected, HFD caused significant increased level of all the detected parameters of CHOL, HDL, LDL, and blood glucose (*p* < 0.05 or *p* < 0.01). While no regulatory effect of lipid metabolism as indicated by TG, CHOL, HDL, LDL, were detected, KSLP significantly reduced the glucose level for HFD mice (14.74 ± 1.74 vs. 10.03 ± 0.56 mmol/L, *p* < 0.05).

**Table 1:** The serum lipid and glucose level in four groups.

	ND	ND_K	HFD	HFD_K
TG (mmol/L)	1.22 ± 0.08	0.88 ± 0.06**	1.02 ± 0.04*	0.94 ± 0.05
CHOL (mmol/L)	2.40 ± 0.10	2.41 ± 0.11	4.71 ± 0.24**	4.12 ± 0.33
HDL (mmol/L)	1.70 ± 0.09	1.67 ± 0.08	2.83 ± 0.03**	2.56 ± 0.21
LDL (mmol/L)	0.30 ± 0.03	0.32 ± 0.01	0.79 ± 0.14*	0.65 ± 0.05
Glucose (mmol/L)	9.60 ± 0.39	7.19 ± 0.53	14.74 ± 1.74*	10.03 ± 0.56^#^

Compared with ND group, *p < 0.05, **p < 0.01. Compared with HFD group, ^#^p < 0.05.

The capacity for glucose metabolism was also evaluated by OGTT on the 9th week of the experiment. As indicated in **Figure 1C**, for each group of mice, the supplementation of glucose resulted in transient increase of blood glucose level at 30 min followed by the gradual decrease of that at 60 and 120 min. HFD mice presented higher levels of glucose at baseline (0 min) and at 30 and 60 min compared with ND mice (*p* < 0.05 or *p* < 0.01). Compared with HFD mice, HFD_K mice showed lower levels of glucose at each indicated time point, and at 30 min, the difference was statistically significant (*p* < 0.05). Similarly, while the AUC of glucose was calculated from the OGTT curve (**Figure 1D**), HFD mice had a significantly higher level of AUC compared to ND mice, and the AUC was marginally decreased in HFD_K group mice compared with HFD mice (**Figure 1D**). The OGTT results suggested that KSLP improved the glucose tolerance disorder in obesity mice.

### KSLP Modulated the Overall Structure of the Gut Microbiota in HFD Mice

**Figure 2:**
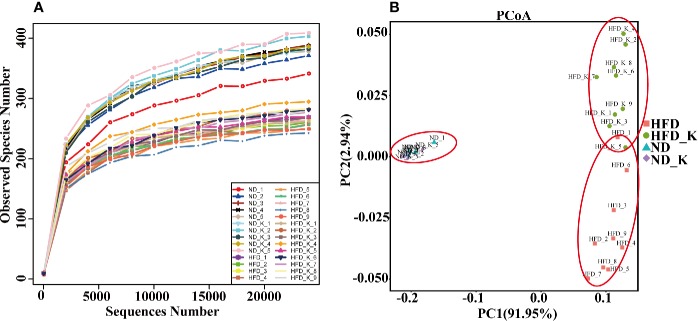
KSLP reconstructed gut microbiota community in HFD mice. **(A)** Rarefaction curve analysis. The abscissa is the number of sequencing randomly extracted from a sample, and the ordinate is the number of OTUs that can be constructed based on the number of sequencing numbers. **(B)** Plots were generated using the principal coordinates analysis (PCoA).

To examine the effect of HFD and KSLP on the gut microbiota, we performed Life ION S5 sequencing targeting the V3–V4 regions of 16S rRNA gene. After selecting the effective reads, a total of 1,256,721 effective reads was generated, and each fecal sample produced an average of 44,883 ± 3769 effective reads. Rarefaction curve analysis reached stable level, indicating that the sequencing depth had covered rare new phylotypes and most of the diversity (**Figure 2A**).

The gut microbiota diversity and richness were evaluated by ACE, chao1, Shannon, and Simpson indexes (**Table 2**). While no obvious difference was noticed between the ND and ND_K group, significant reduced diversity and richness were found in the HFD group compared with the ND group (*p* < 0.01 for all the indexes). Compared with HFD, HFD_K group mice had increased index for all the four parameters, among which the changes in ACE was statistically significant (287.2 ± 3.3 vs. 305.7 ± 4.2, *p* < 0.05). Since obese individuals normally appear with decreased bacterial diversity in the gut (Turnbaugh et al., 2009), our result of KSLP brought up gut microbiota diversity in the HFD mice, indicating that those obese mice improved their body status.

**Table 2:** The effects of HFD and KSLP on gut microbiota diversity.

	ND	ND_K	HFD	HFD_K
ACE	422.2 ± 7.7	443.6 ± 7.8	287.2 ± 3.3**	305.7 ± 4.2^#^
Chao1	436.6 ± 11.7	450 ± 8.1	289.6 ± 3.6**	306.4 ± 3.1
Shannon	6.45 ± 0.06	6.44 ± 0.05	5.64 ± 0.07**	5.85 ± 0.10
Simpson	0.976 ± 0.002	0.972 ± 0.002	0.956 ± 0.003**	0.962 ± 0.004

Compared with ND group, **p < 0.01. Compared with HFD group, ^#^p < 0.05.

UniFrac-based PCoA was assessed to compare the overall microbiota structure for each group (**Figure 2B**). While ND and ND_K clusters merged into one, there appeared distinct clusters for ND/ND_K, HFD, and HFD_K groups. This result suggested that KSLP changed the overall gut microbiota composition in HFD mice but had no effect on ND mice.

### KSLP Regulated Gut Microbiota at Phylum and Family Levels in HFD Mice

**Figure 3:**
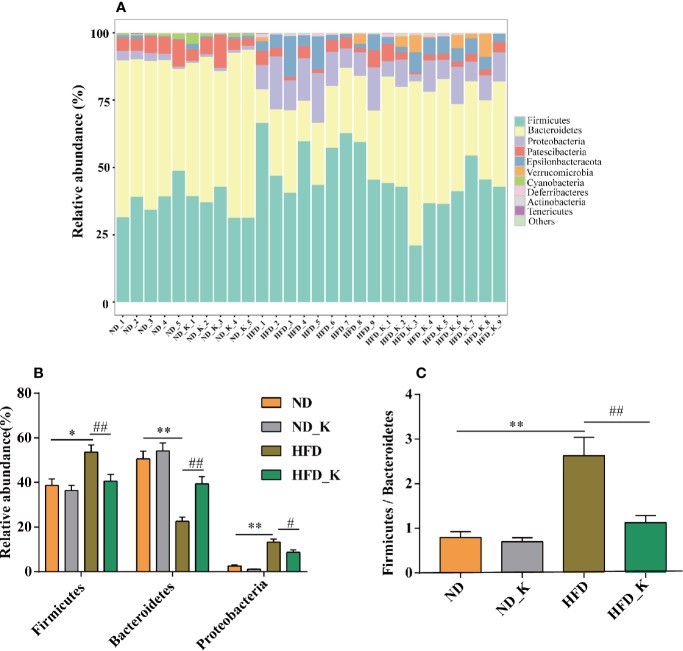
KSLP affected the relative abundance of gut microbiota at the phylum level. **(A)** The relative abundance of the 10 top-ranked phyla were presented. **(B)** The relative abundance of Firmicutes, Bacteroidetes, and Proteobacteria. **(C)** The ratio of Firmicutes/Bacteroidetes. Compared with ND group, *p < 0.05, **p < 0.01. Compared with HFD group, ^#^*p* < 0.05, ^##^*p* < 0.01.

The top 10 phyla in the relative abundance of gut microbiota were shown as histograms (**Figure 3A**), from which Firmicutes, Bacteroidetes, and Proteobacteria were listed as the top three dominant phyla in each group. Further comparison indicated that compared to the ND group, the HFD group had significantly increased relative abundance of Firmicutes and Proteobacteria but reduced relative abundance of Bacteroidetes. Notably, KSLP significantly reversed the phyla changes associated with HFD (*p* < 0.05 or *p* < 0.01, **Figure 3B**). In addition, we compared the ratio of Firmicutes/Bacteroidetes (F/B) as a featured sign of obesity among the four groups. As indicated in **Figure 3C**, HFD significantly increased the ratio of F/B, and its effect could be conversed by KSLP.

**Figure 4:**
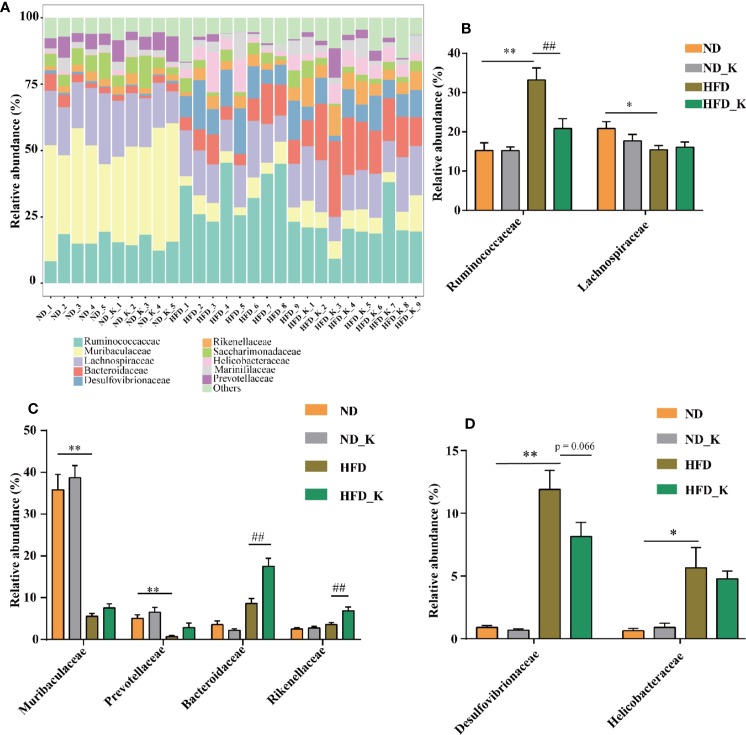
KSLP affected the relative abundance of gut microbiota at the family level. **(A)** The relative abundance in the 10 top-ranked families was presented. **(B)** The relative abundance of Ruminococcaceae and Lachnospiraceae. **(C)** The changed abundance of families under Bacteroidetes phylum. **(D)** The relative abundance of Desulfovibrionaceae and Helicobacteraceae. Compared with ND group, *p < 0.05, **p < 0.01. Compared with HFD group, ^##^*p* < 0.01.

At the family level, in general, as indicated in **Figure 4A** and **Supplementary Table 1**, while ND and ND_K group mice presented very similar gut microbiota composition, HFD significantly affected the relative abundance of certain family and some of them could be reversed by KSLP. In detail, as Ruminococcaceae and Lachnospiraceae are dominant families in the phylum Firmicutes (Chi et al., 2018), HFD resulted in increased abundance of Ruminococcaceae but decreased abundance of Lachnospiraceae (**Figure 4B**). KSLP regulated the abundance of Ruminococcaceae in HFD mice to that similar to ND ones but did not affect that of Lachnospiraceae (**Figure 4B**). Besides, as Muribaculaceae, Prevotellaceae, Bacteroidaceae, and Rikenellaceae are members of the Bacteroidetes phylum, HFD diminished the relative abundance of Muribaculaceae and Prevotellaceae but did not affect that of Bacteroidaceae and Rikenellaceae (**Figure 4C**). Interestingly, KLSP had no impact on the relative abundance of the former two but increased the ones of the latter two (**Figure 4C**). In addition, HFD mice presented enriched Desulfovibrionaceae under Proteobacteria phylum as well as the Helicobacteraceae under Epsilonbacteraeota phylum, and KSLP decreased the abundance of both bacteria at a certain degree (**Figure 4D**).

### KSLP Regulated Gut Microbiota at the Genus Level in HFD Mice

**Figure 5:**
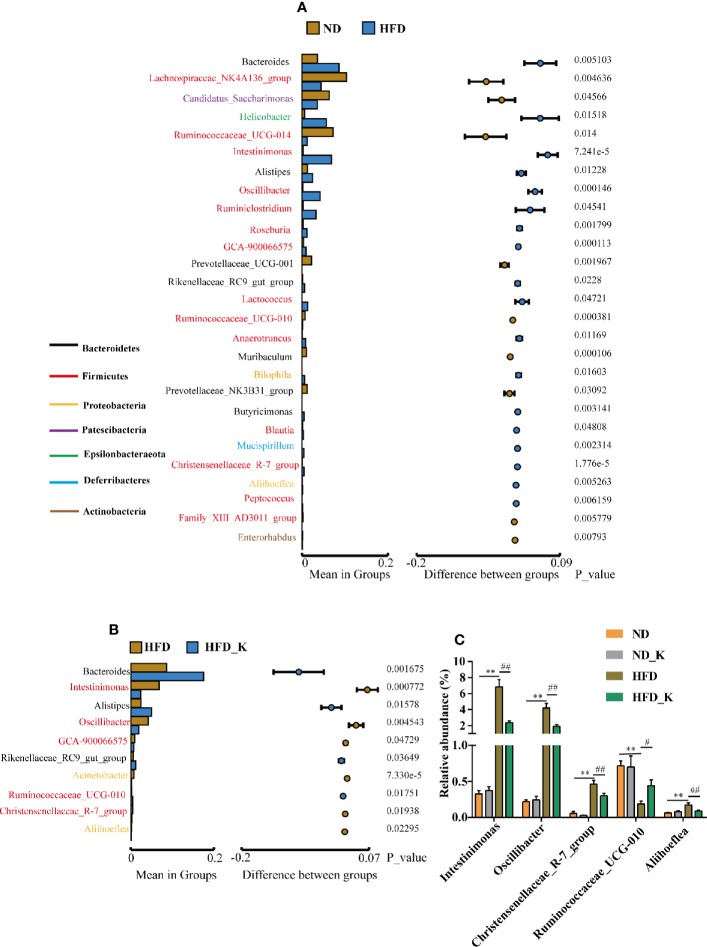
KSLP affected the relative abundance of gut microbiota at the genus level. **(A)** Genera between ND and HFD groups with significant differences were presented. The list of genera with font color refers to their phyla. The histograms presented the mean relative abundance of genera whereas the circular colors represented the differences in confidence intervals. The *p* values for corresponding genus resulting from inter-group significance test were displayed. **(B)** Genera between HFD group and HFD_K group with significant differences were presented. **(C)** The genera responding to HFD and reversed by KSLP treatment. Compared with ND group, **p < 0.01. Compared with HFD group, ^##^p < 0.01, ^#^p < 0.05.

At the genus level, the gut microbiota whose abundance significantly changed due to HFD (ND vs. HFD) and KSLP treatment (HFD vs. HFD_K) were evaluated. The list of the top ranked genera and the significantly changed ones are illustrated in **Figures 5A, B**. Overall, from the 50 top ranked genera in mean abundance between the ND and HFD group, 27 genera were significantly changed in response to HFD (**Figure 5A**). In detail, we observed significantly decreased abundance of the Prevotellaceae_UCG-001, *Muribaculum*, and Prevotellaceae_NK3B31_group of phylum Bacteroidetes in HFD compared with ND mice (**Figure 5A**). Notably, KSLP had no regulatory effect on those bacteria, but alternatively increased the abundance of other genera of bacteria in the category of phylum Bacteroidetes, i.e., Bacteroides, Alistipes, and Rikenellaceae_RC9_gut_group (**Figure 5B**). In addition, compared with the ND group, HFD mice had increased abundance of *Intestinimonas*, *Oscillibacter*, *Ruminiclostridium*, *Roseburia*, *Lactococcus*, Ruminococcaceae_UCG-010, *Anaerotruncus*, *Blautia*, and Christensenellaceae_R-7_group, all of which are of Firmicutes phylum, as well as increased abundance of *Bilophila* and *Aliihoeflea*, both of which are of Proteobacteria phylum (**Figure 5A**). Among them, five genera including *Intestinimonas*, *Oscillibacter*, Ruminococcaceae_UCG-010, Christensenellaceae_R-7_group, and *Aliihoeflea* were significantly reversed by the KSLP treatment in HFD mice (**Figure 5C**).

### Key Phylotypes Responding to the KSLP Treatment in HFD Mice

**Figure 6:**
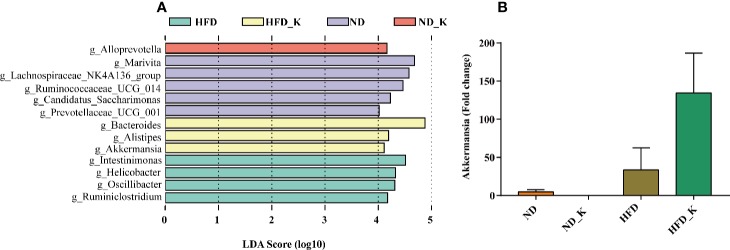
Key phylotypes responding to the KSLP treatment in HFD mice. **(A)** Linear discriminant analysis (LDA) scores were computed for taxa with differential abundance in the fecal microbiota of ND (purple), ND_K (red), HFD (green), and HFD_K (yellow) mice. The LDA score indicated the effect size and ranking of each differentially abundant taxon (LDA > 4). **(B)** Real-time qPCR analysis. The relative expression of Akkermansia was expressed as the fold change in comparison to the ND group.

We also performed LEfSe analysis to identify the specific bacteria in the genus that were characteristic among the four groups. As shown in **Figure 6A**, discriminative features were identified with LDA score > 4.0. Referring to the ranked bacterial taxa, ND mice were enriched with g_Marivita, g_Lachnospiraceae_NK4A136_group, and g_Ruminococcaceae_UCG_014; ND_K mice with g_Alloprevotella; HFD mice with g_Intestinimonas, g_Helicobacter, g_Oscillibacter, and g_Ruminiclostridium; and HFD_K mice with g_Bacteroides, g_Alistipes, and g_Akkermansia. Of note, two of the four phylotypes of HFD, namely, *Intestinimonas* and *Oscillibacter*, were responsible for KSLP treatment (**Figure 5C**). We also noticed that Akkermansia was one of the characteristic genera for HFD_K group mice. The expression of Akkermansia was further analyzed using real-time qPCR analysis. The results demonstrated that compared with the HFD group, the expression level of Akkermansia in the HFD_K group was indeed increased (**Figure 6B**). Previous researches reported that the application of metformin resulted in slowing down body weight gain, improving glucose homeostasis, as well as enrichment of Akkermansia in diet-induced obese mice (Shin et al., 2014; Anhe et al., 2015). Besides, oral administration of Akkermansia (i.e., as a probiotic) could improve HFD induced metabolic disorders (Everard et al., 2013). Therefore, it can be considered that the beneficial effect of KSLP on HFD mice is related to enriched abundance of Akkermansia.

### KSLP Regulated Obesity-Related Gut Microbiota

**Figure 7:**
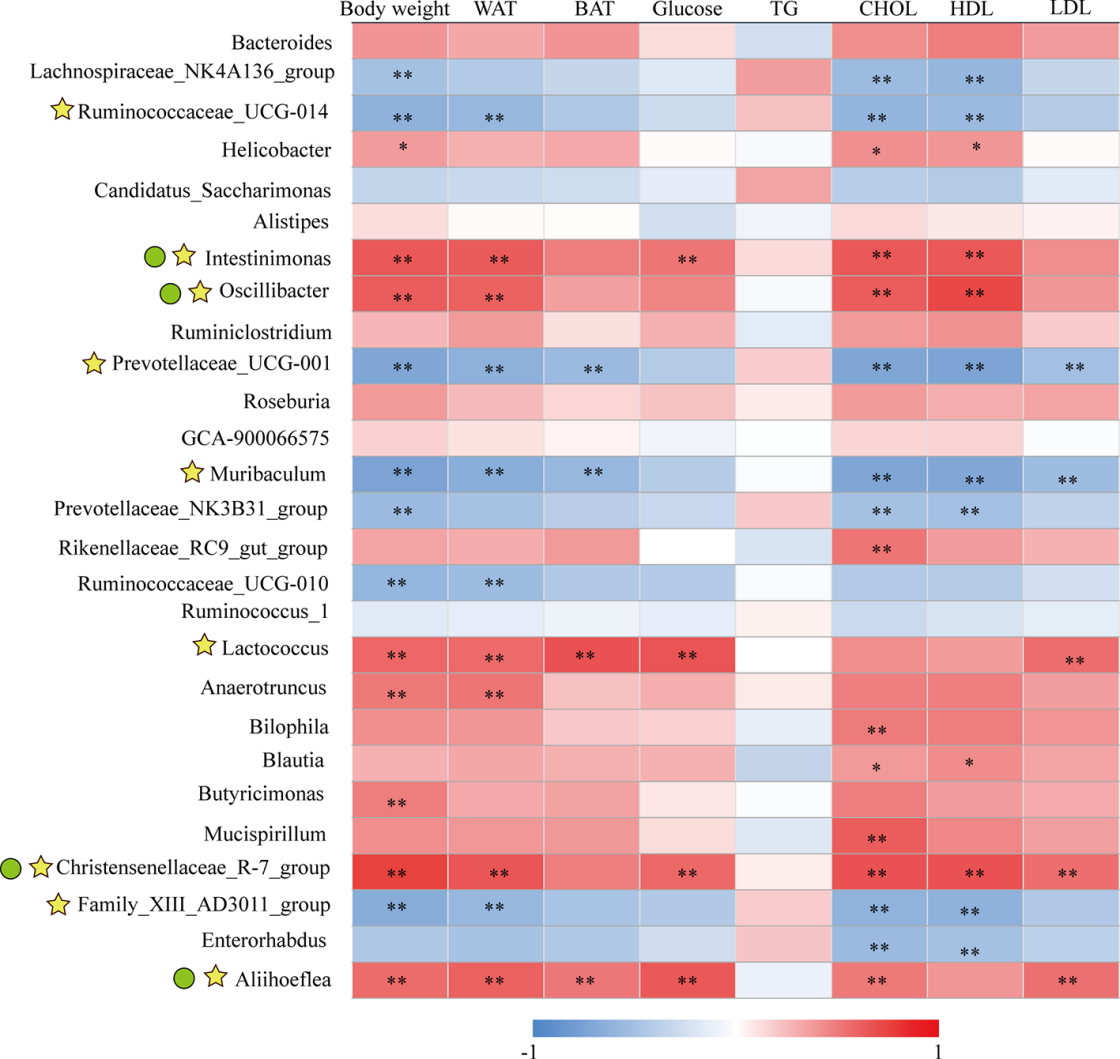
The correlation between the changed genera responding to HFD and obesity-related index.*|*r*| > 0.5 and *p* < 0.05; **|*r*| > 0.5 and *p* < 0.01. Yellow asterisk: ≥4 obesity-related index were intimately correlated with certain genus; green cycle: (1) ≥4 obesity-related index were intimately correlated with certain genus; (2) reversed by KSLP treatment.

As we have identified 27 genera that had significant abundance changes between ND and HFD groups, the correlation between these 27 genera and obesity-related parameters was calculated by Pearson correlation analysis. The result was summarized in **Supplementary Table 2** and presented as a heatmap (**Figure 7**). In general, there were nine genera intimately related to obesity phenotype, from which strong positive co-relationships were identified for *Intestinimonas*, *Oscillibacter*, *Lactococcus*, Christensenellaceae_R-7_group, and *Aliihoeflea*, while significant negative co-relationships were identified for Ruminococcaceae_UCG-014, Prevotellaceae_UCG-001, *Muribaculum*, and Family_XIII_AD3011_group (**Figure 7**). Of these nine genera, *Intestinimonas*, *Oscillibacter*, Christensenellaceae_R-7_group, and *Aliihoeflea* could be reversed by KSLP in HFD mice (**Figure 5C**). Therefore, *Intestinimonas*, *Oscillibacter*, Christensenellaceae_R-7_group, and *Aliihoeflea* might be targets of intervention for KSLP in HFD mice.

### KSLP Modulated Fecal Metabolomic Profiling in HFD Mice

**Figure 8:**
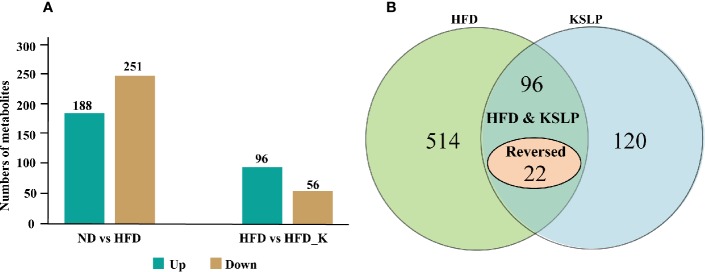
Different metabolites among three groups. **(A)** Numbers of upregulated and downregulated metabolites. **(B)** The influencing factor of metabolites using two-way ANOVA.

We also conducted a global metabolomic profiling analysis using lyophilized fecal samples to check the metabolic situation of the gut in ND, ND_K, HFD, and HFD_K mice. In general, we have identified a total of 775 compounds of known identity (**Supplementary Table 3**). There were 439 metabolites changed in response to HFD, in which 188 were upregulated and 251 were downregulated (**Figure 8A**). Compared with the HFD group, 152 compounds changed, including 96 metabolites upregulated and 56 metabolites downregulated in the HFD_K group (**Figure 8A**). Two way-ANOVA analysis indicated that 514 metabolites could be affected by HFD and 120 metabolites could be affected by KSLP. Furthermore, 96 metabolites could be affected by both HFD and KSLP, in which the contents of 22 metabolites in response to HFD treatment could be reversed by KSLP (**Figure 8B** and **Supplementary Tables 4** and **5**).

### KSLP Regulated Obesity-Related Genera Along With Their Correlated Metabolites

Special attention was paid to reveal the novel inner relationship between the identified obesity-related gut microbiota and the reversed metabolites in response to KSLP in HFD mice. In this regard, we performed a correlation analysis by applying the four identified KSLP-responsible-obesity-related genera of *Intestinimonas*, *Oscillibacter*, Christensenellaceae_R-7_group, and *Aliihoeflea*, as well as the 22 KSLP-responsible-HFD-related metabolites (**Supplementary Table 6**).

**Figure 9:**
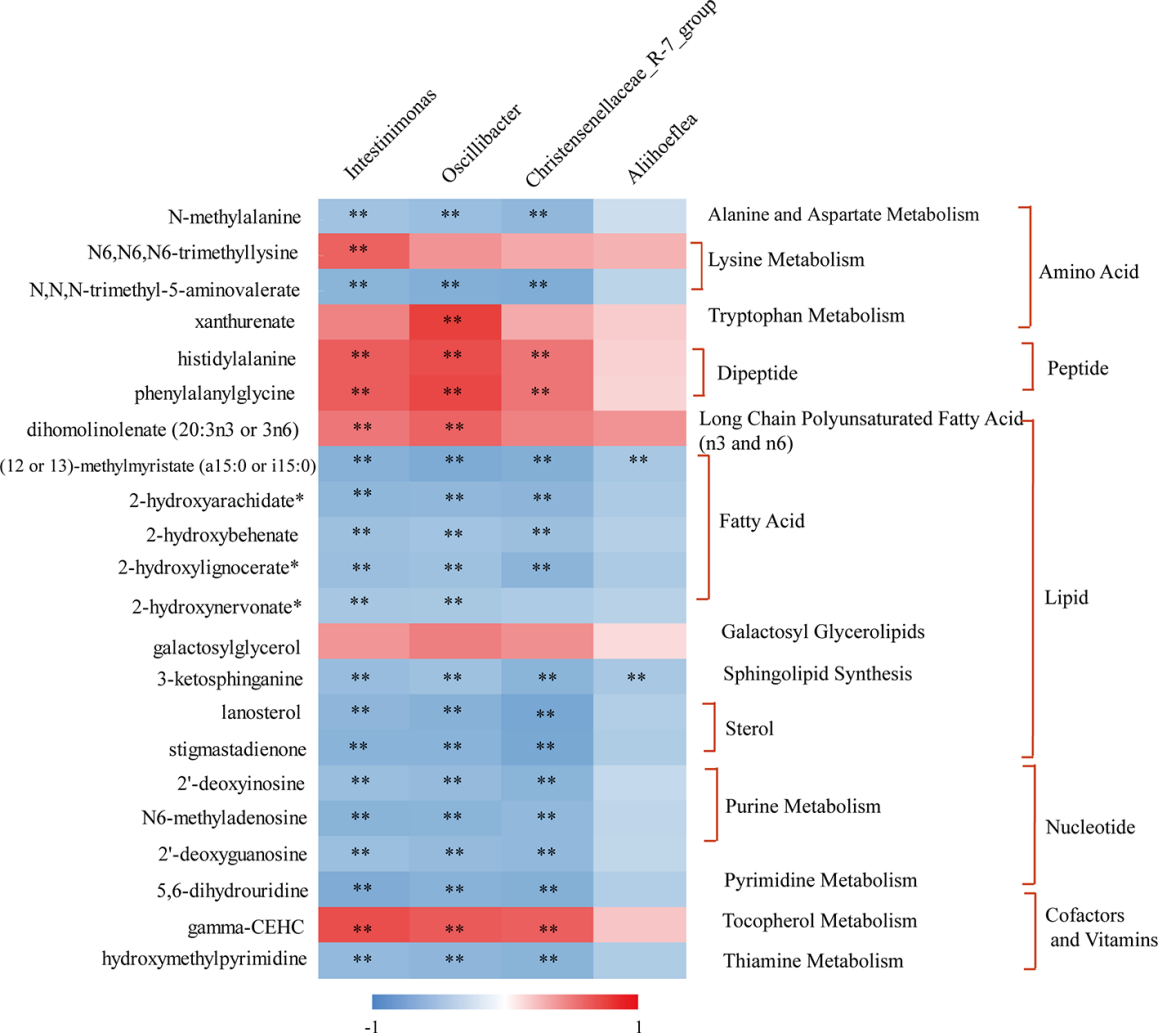
The correlation between KSLP-responsible-obesity-related genera and reversed metabolites by KSLP. **|*r*| > 0.5 and *p* < 0.01. The metabolites corresponding to the metabolic pathway were presented on the right.

As indicated in the heatmap (**Figure 9**), *Intestinimonas*, *Oscillibacter*, and Christensenellaceae_R-7_group showed very similar metabolite relationship patterns, which positively correlated with histidylalanine, phenylalanylglycine, and gamma-CEHC, but negatively correlated with *N*-methylalanine, *N*,*N*,*N*-trimethyl-5-aminovalerate, (12 or 13)-methylmyristate (a15:0 or i15:0), 2-hydroxyarachidate*, 2-hydroxybehenate, 2-hydroxylignocerate*, 3-ketosphinganine, lanosterol, stigmastadienone, 2′-deoxyinosine, N6-methyladenosine, 2′-deoxyguanosine, 5,6-dihydrouridine, and hydroxymethylpyrimidine. As for *Aliihoeflea*, it presented weak correlation with the metabolites in the list except for the negative co-relationships with (12 or 13)-methylmyristate (a15:0 or i15:0) and 3-ketosphinganine.

## Discussion

In this study, we observed significant differences in gut microbiota communities between the ND group and HFD group. At the phyla level, the gut of HFD-fed mice was characterized by increased abundance of Firmicutes and Proteobacteria, decreased abundance of Bacteroidales, as well as increased ratio of F/B, the profiling of which appeared similar to previously reported “obese gut” (Alard et al., 2016; Yu et al., 2018; Zhang et al., 2019b) and obesity-driven gut dysbiosis (Anhe et al., 2019; Jeong et al., 2019). In general, both Firmicutes and Bacteroidales are relevant with energy metabolism homeostasis (Ley et al., 2005). Take, for example, increased abundance of Firmicutes is believed to be beneficial for obese individuals to harvest energy from food, subsequently absorb calories, and eventually gain body weight (Turnbaugh et al., 2006). Since KSLP alleviated the enrichment of Firmicutes, it is reasonable that KSLP could reduce the weight gain in HFD mice without interfering with their food intake.

At the family level, KSLP reversed the appearance of increased abundances of Ruminococcaceae and Desulfovibrionaceae in HFD mice. Ruminococcaceae, as a member of phylum Firmicutes, has been reported to be negatively correlated with hepatic encephalopathy (Bajaj et al., 2012), alcoholic cirrhosis (Bajaj et al., 2014), non-alcoholic fatty liver disease (Schnabl and Brenner, 2014), and intestinal leaking (Leclercq et al., 2014). In obesity-prone mice, the enriched Ruminococcaceae was revealed to be responsible for promoting fat synthesis (Duca et al., 2014). Our results showed that KSLP could reduce the abundance of Ruminococcaceae; therefore, it might be beneficial for reducing fat synthesis initiated by HFD. Desulfovibrionaceae, as a member of the Proteobacteria phylum, is known to be related to the production of endotoxin (Perez-Matute et al., 2015) and inflammation (Xiao et al., 2014). Our results of enriched Desulfovibrionaceae in HFD mice could be considered as a sign for the presence of gut inflammation, and KSLP might help in alleviating the occurrence of inflammation.

Other changes that happened in HFD mice at the family level were decreased abundance of Muribaculaceae and Prevotellaceae, which accounted for the decreased abundance of Bacteroidetes phylum. Interestingly, we noticed that although KSLP could effectively increase the overall abundance of Bacteroidetes phylum, it did not affect the abundance of Muribaculaceae and Prevotellaceae. Alternatively, it did increase two other family members of Bacteroidetes phylum: Bacteroidaceae and Rikenellaceae. Our result suggested that different to its effects on phyla Firmicutes and Proteobacteria, KSLP regulated the functional rather than compositional changes for family bacteria under the category of Bacteroidetes phylum.

At the genus level, Pearson correlations analysis between obesity-related parameters and the 27 changed genera responsible to HFD treatment verified nine obesity-associated genera, among which the abundance of *Intestinimonas*, *Oscillibacter*, Christensenellaceae_R-7_group, and *Aliihoeflea* could be reversed by KSLP. Notably, the roles of *Intestinimonas* and *Oscillibacter* in obesity have been reported. For instance, *Intestinimonas* is generally considered to be a beneficial genus with butyrate-producing capacity (Yang et al., 2019); nevertheless, the enrichment of which also presents in HFD rats (Lin et al., 2019). As for *Oscillibacter*, it is revealed to be positively correlated with the occurrence of obesity (Chang et al., 2015; Chang et al., 2017) probably through regulating fat synthesis (Duca et al., 2014). Until now, few studies revealed the roles of Christensenellaceae_R-7_group and *Aliihoeflea* in obesity. It seems that carnivorous individuals harbor abundance of Christensenellaceae_R-7_group, assigned to the Firmicutes phylum (Huang, 2018). Besides, destined as pathological Proteobacteria, the appearance of *Aliihoeflea* could be recognized as a sign reflecting gut disorder during the course of dextran sodium sulfate (DSS)-triggered colitis (Zhang et al., 2019a). In view of the above evidences, we believe that *Intestinimonas*, *Oscillibacter*, Christensenellaceae_R-7_group, and *Aliihoeflea* play crucial roles regarding the prevention effect of KSLP against HFD-induced obesity.

Gut microbiota affects host metabolism by ways of transformation, absorption, and metabolism of exogenous substances (Li et al., 2019). It is rational that the alternation of metabolites reflects the changes of gut microbiota profiles. In the current study, we performed Pearson correlation analysis to reveal the co-relation between the four identified obesity-related genera and the metabolites responding to KSLP. We found that there existed very similar metabolite relationship patterns of *Intestinimonas*, *Oscillibacter*, and Christensenellaceae_R-7_group. As summarized in **Figure 9**, those metabolites could be functionally categorized into amino acid, dipeptide, lipid metabolism, and others. Amino acids are believed to play critical roles in maintaining host gut homeostasis since their disorder happens in germ-free mice (Whitt and Demoss, 1975). The compositional changes of obesity-related gut microbiota may affect the production of certain amino acids as by-products of detrimental bacteria (Bested et al., 2013). Interestingly, it was recently revealed that xanthurenic acid and other products of tryptophan metabolism are related to the production and releasing of insulin (Oxenkrug, 2013). In our study, HFD mice had significantly increased level of xanthurenate in the gut. This, together with the fact that HFD mice appeared with poor glucose tolerance capacity, indicated that there might exist a correlation of HFD/gut microbiota/xanthurenate/glucose tolerance pipeline. Since the application of KSLP not only decreased the gut xanthurenate level, but also improved glucose tolerance capacity and reduced blood glucose level in HFD mice, a stimulated insulin production could be expected. Notably, there was no strong direct link between *Oscillibacter* itself and glucose (**Figure 7**); it is likely that a panel of microbiota, i.e., *Oscillibacter*, by working together with *Intestinimonas* or Christensenellaceae_R-7_group, functionally affects glucose metabolism. We also noticed that the content of fecal lanosterol was negatively related to the abundance of *Intestinimonas*, *Oscillibacter* and Christensenellaceae_R-7_group. Lanosterol is a precursor of cholesterol synthesis (Zhao et al., 2015), the reduced production of which in feces might be indicated for an increased cholesterol level in the serum. In HFD mice, supplementation with KSLP resulted in higher lanosterol than in feces of HFD mice alone; therefore, KSLP might impact sterol absorption in the intestine. Although not well defined, there are reports that highlight the regulatory roles of peptides and fatty acids in the scenario of obesity and its related metabolic disease (Zhao, 2011; Miyamoto et al., 2019). Taken together, our data suggest that KSLP may influence the abundance of *Intestinimonas*, *Oscillibacter*, and Christensenellaceae_R-7_group, which affects the fecal metabolites and whole-body physiology in HFD mice.

## Conclusions

In conclusion, our study provides sufficient evidences showing that KSLP is a promising TCM with prevention effects against HFD-induced obesity and is applicable for its consequent occurrence of metabolic diseases. The mode of action of KSLP is most likely *via* modulating the gut microbiota. As a result, the obesity-related gut dysbiosis including the decreased diversity, increased F/B ratio, and enriched abundance of Proteobacteria could be corrected. *Intestinimonas*, *Oscillibacter*, and Christensenellaceae_R-7_group might be responsible for the regulatory effect of KSLP on obesity-related metabolic disorders, which involve amino acid, peptide, and lipid metabolism. In addition to the verification of the inner links among obesity, gut microbiota, and metabolic disorders, further investigations are required to verify the key targets of intervention as well as bioactive compounds in KSLP.

## Data Availability Statement

The datasets generated for this study can be found in the PRJNA565488.

## Author Contributions

JQ, HC, and MY conceived and designed the study. SG, MXW, TY, and MYW performed most of the experiments. SG, YL, LM, and YY analyzed and interpreted the data. LL provided the technical support. SG, JQ, and YW drafted the article. XZ contributed the constructional suggestions for revision. All authors made the final approval of the version to be submitted.

## Funding

This work was supported by grants from the National Key Scientific and Technological Project of China (2019ZX09201005) and the Science and Technology Department of Zhejiang Province of China (2018C02048).

## Conflict of Interest

Author LL was employed by company Chiatai Qingchunbao Pharmaceutical Co. Ltd.

The remaining authors declare that the research was conducted in the absence of any commercial or financial relationships that could be construed as a potential conflict of interest.

## Supplementary Material

Supplementary Table 1 The relative abundance of the top 10 families.

Supplementary Table 2 Pearson correlation analysis between obesity-related parameters and genera.

Supplementary Table 3 The effects of HFD and KSLP supplement on Mice feces metabolites.

Supplementary Table 4 The metabolites in response to both the factors of HFD and KSLP.

Supplementary Table 5 The metabolites in response to both the factors of HFD and KSLP, in which can be reversed by KSLP.

Supplementary Table 6 The correlation coefficient between key genera and reversed metabolites.
