# Supplementary material for: Traditional Chinese Medicine Formula Kang Shuai Lao Pian Improves Obesity, Gut Dysbiosis, and Fecal Metabolic Disorders in High-Fat Diet-Fed Mice
Source: Front Pharmacol. 2020 Mar 25;11:297. doi: 10.3389/fphar.2020.00297 (PMC7109517; doi:10.3389/fphar.2020.00297)
Supplement: Supplementary file 7 [file DataSheet_1.pdf]

Supplementary Figures

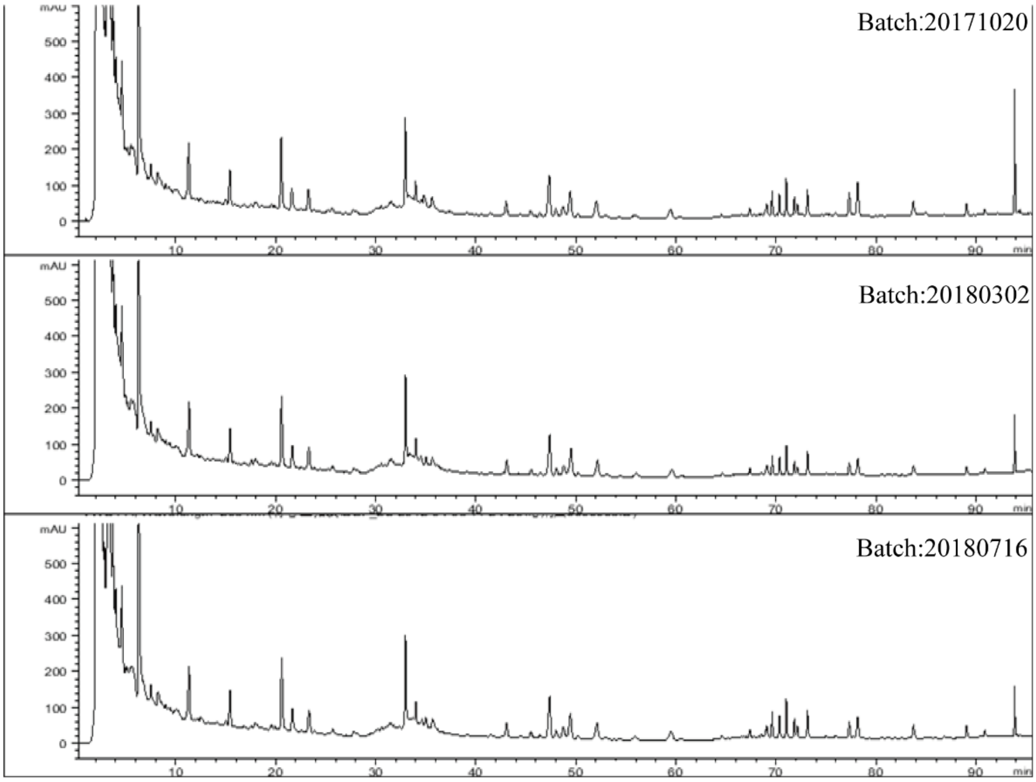

Suppl. Figure 1 HPLC fingerprint of three batchs KSLP sample. Ultrasonic extraction (power 250 W, frequency 40 Hz) was used to sample preparation for HPLC fingerprint. Briefly, KSLP were ground, then an accurately weighed sample (2 g) of the fine powder was extracted with 45 % methanol (40 mL) for 60 min, respectively. The mixture was filtered, and the filtrate was evaporated. The residue was dissolved in 14 % acetonitrile aqueous solution, then transferred to a 10 mL volumetric flask, next added 14 % acetonitrile aqueous solution to tick mark. The sample solutions were filtered through a 0.45 μm membrane before HPLC analysis. The analysis was performed on an Agilent 1200 series HPLC system (Agilent, Waldbronn, Germany), equipped with quaternary pump with online degasser, temperature-controlled autosampler. The samples were separated on an Agilent Eclipse Plus C18 column (4.6 × 50 mm, 1.8 μm). The mobile phase was composed of acetonitrile solution (A) and water (B) and the gradient elution program was as follows:88%-85% (B) for 0–8 min; 85–81% (B) for 8–18 min; 81–80% (B) for 18–25 min; 80–73% (B) for 25–30 min; 73–68% (B) for 30–45 min; 68–67% (B) for 45–60 min; 67–45% (B) for 60–70 min; 45–40% (B) for 70–80 min; 40–35% (B) for 80–85 min; 35–10% (B) for 85–90 min; 10–0% (B) for 90–92 min. The injection volume was 20 μL; the flow rate was 0.8 mL/min; and the column temperature was 25°C. The detector was set to 203 nm.

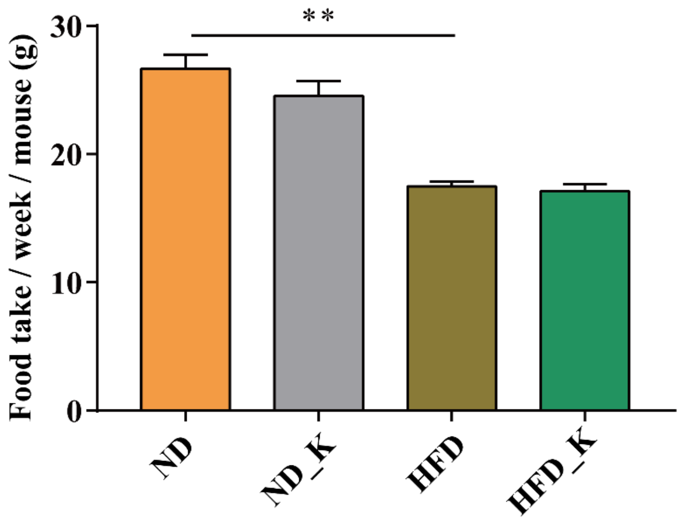

Suppl. Figure 2 The mean food take of each mouse in a week. ** compared with ND group, $p < 0.01$

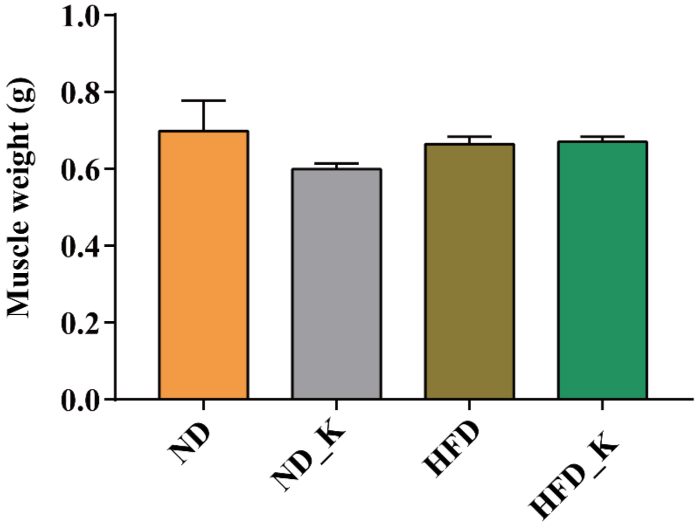

Suppl. Figure 3 The effects of HFD and KSLP on muscle weight.
